# Supplementary material for: Eligibility for hepatitis B antiviral therapy among adults in the general population in Zambia
Source: PLoS One. 2020 Jan 13;15(1):e0227041. doi: 10.1371/journal.pone.0227041 (PMC6957183; doi:10.1371/journal.pone.0227041)
Supplement: Attachment — Submitted filename: Comments_schweitzer_191012.docx [file pone.0227041.s001.docx]

Comments: Manuscript Number **PONE-D-19-24241**

The authors have reported on the eligibility for hepatitis B antiviral therapy among adults in the general population in Zambia. The authors may wish to consider the following comments:

1. The authors report, “No HBsAg-positive individual reported signs or symptoms of acute infection”. Well, many newly HBV infected people have no symptoms at all. So, this is not a very rigorous criterion to exclude acute infections. Why was the HBc antibody (IgM anti-HBc) measured in N = 580? Could you please provide some details in your flow chart?
2. “HIV coinfection **was common**, with 26 (14.4%) HBsAg.” I would suggest that the authors tone this down a bit. The description “relatively high” might more fitting.
3. “Pregnant women were more likely than non-pregnant women to be HBsAg-positivity 5.5% versus 3.0%), but the difference was not statistically significant after adjustment for age (P=0.06)”. The study might be underpowered to make this assessment, (only N = 8 women were pregnant); the result might have been statistically significant with a higher sample size. Besides, P= 0.06 is pretty close to the significance level. I would address this obvious limitation in the text. Moreover, did the authors consider the following covariates: the number of previous deliveries, and hospital stay, which might confound the relationship?
4. An important limitation of the study regarding correlates of HBsAg is missing confounder bias. This should be adequately addressed in the text.
5. The authors mention the higher odds of being HBsAg positive and higher odds of infectivity and state that “Men should be a major focus of HBV elimination strategies in Africa”, but what are the consequences of this for women and children? I am not sure how they propose to do this given the reported gender-based disparity in access to healthcare that already exists in developing countries. The authors address correlates of HBV infection, but fail to take marital status into account.
6. The authors state that “The odds of HBsAg-positivity among 30- to 39-year-olds was significantly higher than 18- to 19-year-olds” and give some explanations in this regard including public health interventions etc. But, the birth cohort effect has not been sufficiently addressed here. Sexual transmission and infections including reinfections in adulthood also contribute to this and should be mentioned.
7. Chronic HBV infections are usually asymptomatic so I am not quite sure if I understand what the authors are trying to say in the following sentence: “HBV infection in the general population in Africa is usually asymptomatic as none of the HBsAg positive adults assessed had signs of decompensated cirrhosis”. It is because chronic HBV infection is symptomless, that it is also referred to as a silent epidemic.
8. The authors state, “Income and location (urban versus rural) were not associated with HBsAg”. The authors have not addressed (internal and external) migration patterns in their analysis, specifically in their interpretation. Could this have influenced the recorded prevalence?
9. The authors report “Sociodemographic correlates of HBsAg-positivity were identified”. However, the risk factors that have been considered in the analysis are not comprehensive. This should be addressed in the limitations of the study.
10. I am missing a detailed description of the statistical analysis, including model fit statistics in the methods section. Were interactions looked for?
11. Was multiple-comparison testing done in the adjusted regression models shown in Table 1?
12. What was the R^2^ of the fully adjusted logistic regression model? How much variation was explained by the fully adjusted logistic regression model?
13. The authors report that “HIV coinfection was noted in 14% of HBsAg-positive individuals and the majority were taking tenofovir-containing antiretroviral therapy (ART).” If this is indeed the case, then what are the ramifications for HBV treatment programs in Africa? In resource-poor settings with overlapping HIV and HBV epidemics one could embed HBV elimination programs in running HIV programs. This should be addressed in brief in the discussion.
14. What are the authors’ recommendations in terms of the available guidelines that should be use in Africa based on their analysis/findings? Some comment in this regard would be helpful.

**Minor comments:**

Figure 1: In the flowchart the abbreviation “DBS” has been used without the full phrase being used anywhere in the text.
