# Supplementary material for: Eligibility for hepatitis B antiviral therapy among adults in the general population in Zambia
Source: PLoS One. 2020 Jan 13;15(1):e0227041. doi: 10.1371/journal.pone.0227041 (PMC6957183; doi:10.1371/journal.pone.0227041)
Supplement: Attachment — Submitted filename: Response to reviewers.docx [file pone.0227041.s002.docx]

**Point by point responses to reviewer comments**

**Review comment: The authors report, “No HBsAg-positive individual reported signs or symptoms of acute infection”. Well, many newly HBV infected people have no symptoms at all. So, this is not a very rigorous criterion to exclude acute infections. Why was the HBc antibody (IgM anti-HBc) measured in N = 580? Could you please provide some details in your flow chart?**

Our response: We agree that lack of symptoms cannot rule out acute HBV and we have removed this line from the revised manuscript. We originally attempted to measure HBc in dried blood spots collected in the communities where participants were recruited. However, the assay’s accuracy on DBS was too poor, so we decided to remove these data from the paper. In the revised flow chart (pasted in below), we have removed any mention of anti-HBc testing.

**Reviewer comment: “HIV coinfection was common, with 26 (14.4%) HBsAg.” I would suggest that the authors tone this down a bit. The description “relatively high” might more fitting.**

Our response: We agree and have made the suggested revision.

**Reviewer comment: “Pregnant women were more likely than non-pregnant women to be HBsAg-positivity 5.5% versus 3.0%), but the difference was not statistically significant after adjustment for age (P=0.06)”. The study might be underpowered to make this assessment, (only N = 8 women were pregnant); the result might have been statistically significant with a higher sample size. Besides, P= 0.06 is pretty close to the significance level. I would address this obvious limitation in the text. Moreover, did the authors consider the following covariates: the number of previous deliveries, and hospital stay, which might confound the relationship?**

Our response: To clarify, 217 pregnant (12 were positive) and 2,815 non-pregnant women (85 were positive) were tested for HBsAg. Only 8 pregnant women with HBsAg were comprehensively evaluated at the hospital. While repeating our multivariable analysis of correlates of HBsAg-positivity, we included pregnancy status. A trend towards higher HBsAg-positivity in pregnant versus non-pregnant women was seen, although it did not reach statistical significant and hence could be due to chance alone.

In the revised manuscript, we briefly discussed this.

In results (page 9, paragraph 3), we state *In bivariable analysis, HBsAg-positivity was slightly higher in men compared to women (4.2% versus 3.3%; P=0.12) and among pregnant versus non-pregnant women (5.5% versus 3.0%; P=0.04). Interactions between age and sex and sex and income were not statistically significant. In multivariable analysis, participants aged 30-39 had 2.11 times the odds of HBsAg-positivity (95% CI, 0.96-4.63) compared to those aged 18-19 years. Men (adjusted odds ratio [AOR], 1.37; 95% CI, 0.99-1.89) and pregnant women (AOR, 1.74; 95% CI, 0.93-3.25) had trends toward increased HBsAg-positivity compared to non-pregnant individuals.*

We stated (page 16, paragraph 2), *In our analysis, pregnant women also had a trend toward increased HBsAg compared to non-pregnant women, which could be due to immune suppression that occurs in pregnancy. None of the 8 further evaluated met guidelines for antiviral therapy for PMTCT. Further analysis of this is warranted as our sample size of pregnant women (n=217) was small.*

**Reviewer comment: An important limitation of the study regarding correlates of HBsAg is missing confounder bias. This should be adequately addressed in the text.**

Our response: Unmeasured confounders are an inherent limitation of all observational analyses including this one. In the revised manuscript under limitations we now mention (page 17, paragraph 2) that, “only a limited number of sociodemographic factors were evaluated.”

**Reviewer comment: The authors mention the higher odds of being HBsAg positive and higher odds of infectivity and state that “Men should be a major focus of HBV elimination strategies in Africa”, but what are the consequences of this for women and children? I am not sure how they propose to do this given the reported gender-based disparity in access to healthcare that already exists in developing countries.**

Our response: Thank you for this question. At the end of the day, the way viral hepatitis kills people is by causing liver cancer and cirrhosis. Liver cancer (the majority due to HBV) is the 1^st^ or 2^nd^ most common cancer in men in Africa. Also, it is well established that men are more likely than women to develop these outcomes if they are HBsAg-positive, due to the protective effects of estrogen, due to more environmental exposures in men, and perhaps other mechanisms. We agree that women may face disparities in access to healthcare compared to men. However, with certain infections like HIV and TB, reaching men is more difficult because they do not attend the health facilities and have lower rates of retention in chronic care compared to women. We just believe that men should be a priority group as they bear the majority of the mortality burden. We revised the above sentence (page 15, paragraph 2) to read, *“As HBV elimination strategies are implemented in Africa, these data suggest that men should be a priority population.“*

**Reviewer comment: The authors address correlates of HBV infection, but fail to take marital status into account.**

Our response: We have revisited our analysis of correlates of HBsAg-positivity. As suggested, we included marital status (never versus ever married). Upon further consideration, we also decided to use income level as a continuous variable rather than categorizing it as we did in the original paper. The revised table is presented below. It shows that being married was not associated with HBsAg-positivity.

|  | Unadjusted  OR (95% CI) | Adjusted  OR (95% CI) |
| --- | --- | --- |
| Age, in years  18-19  20-29  30-39  40-49  50-59  ≥60 | Reference  1.43 (0.73-2.80)  2.16 (1.10-4.25)  1.51 (0.73-3.12)  1.03 (0.44-2.48)  0.26 (0.07-0.95) | Reference  1.42 (0.70-2.87)  2.11 (0.96-4.63)  1.44 (0.61-3.38)  1.06 (0.41-2.78)  0.26 (0.06-1.05) |
| Sex  Female  Male | Reference  1.26 (0.94-1.71) | Reference  1.37 (0.99-1.89) |
| Currently pregnant  No  Yes | Reference  1.62 (0.89-2.96) | Reference  1.74 (0.93-3.25) |
| Marital status  Never married  Ever married | Reference  1.09 (0.78-1.53) | Reference  0.99 (0.63-1.53) |
| Household income, per 1 item* | 0.90 (0.82-0.98) | 0.89 (0.81-0.98) |
| Location of current residence  Rural  Urban | Reference  0.82 (0.58-1.17) | Reference  0.74 (0.52-1.06) |
| *Based on a 10-item household possession index  Abbreviations: OR, odds ratio; CI, confidence interval | | |

**Reviewer comment: The authors state that “The odds of HBsAg-positivity among 30- to 39-year-olds was significantly higher than 18- to 19-year-olds” and give some explanations in this regard including public health interventions etc. But, the birth cohort effect has not been sufficiently addressed here. Sexual transmission and infections including reinfections in adulthood also contribute to this and should be mentioned.**

Our response: We now revise the term time trends to cohort effect (page 16, paragraph 2). The paragraph on HBsAg-positivity by age now reads as follows:

*This could be due to cohort effects (i.e., changes in behavior or in other public health interventions phased in over time); however, several studies in Africa have suggested that sexual transmission in adulthood may contribute new HBV infections. Sexual risk behavior was linked with HBV infection in Uganda [**30**], and adults taking HBV-active antiretroviral therapy for HIV had reduced HBV incidence [**31**]. In homosexual Kenyan men, recent HIV acquisition and rape were associated with higher HBcAb incidence while circumcision was associated with decreased risk [**32**]. Although preventing vertical and early childhood transmission, when the risk for chronic infection is highest, remains the priority, a better understanding of sexual transmission of HBV in Africa is needed.*

**Reviewer comment: Chronic HBV infections are usually asymptomatic so I am not quite sure if I understand what the authors are trying to say in the following sentence: “HBV infection in the general population in Africa is usually asymptomatic as none of the HBsAg positive adults assessed had signs of decompensated cirrhosis”. It is because chronic HBV infection is symptomless, that it is also referred to as a silent epidemic.**

Our response: While the review is a hepatitis expert, many of the readers of *Plos One* are not, which is why we wanted to include this comment. If the editor prefers, we can delete this line altogether.

**Reviewer comment: The authors state, “Income and location (urban versus rural) were not associated with HBsAg”. The authors have not addressed (internal and external) migration patterns in their analysis, specifically in their interpretation. Could this have influenced the recorded prevalence?**

Our response: We agree that migration between urban and rural areas, which is common, creates noise in the analysis (i.e., many urban dwellers may have grown up and been exposed to HBV in rural areas before migrating to the city). We did not collect any data on where participants were born (urban versus rural), which could have strengthened the analysis. In the revised table 1 (see above), we labelled the variable, “location of current residence” to acknowledge this issue.

**Reviewer comment: The authors report “Sociodemographic correlates of HBsAg-positivity were identified”. However, the risk factors that have been considered in the analysis are not comprehensive. This should be addressed in the limitations of the study.**

Our response: In the revised discussion section, under limitations, we have added comment (page 17, paragraph 2) that “*Only a limited number of sociodemographic factors were evaluated*.”

**Reviewer comment: I am missing a detailed description of the statistical analysis, including model fit statistics in the methods section. Were interactions looked for? Was multiple-comparison testing done in the adjusted regression models shown in Table 1? What was the R^2^ of the fully adjusted logistic regression model? How much variation was explained by the fully adjusted logistic regression model?**

Our comment: All covariates included in the final model were identified *a priori*. Interactions were explored though they did not make the cutoff for model inclusion (page 8, paragraph 1). We did not evaluate an R-squared value give the potentially unforeseen level of variation across the variables included. Our aim was to describe associations, not to produce predictions.

As requested, in the revised manuscript, we added more details on our statistical analysis. On page 8, we added, *“Confounders included in the final model were identified a priori. Interaction between age and sex, and sex and income were considered for inclusion using a cutoff p-value of 0.2 in the bivariate analysis.“*

**Reviewer comment: The authors report that “HIV coinfection was noted in 14% of HBsAg-positive individuals and the majority were taking tenofovir-containing antiretroviral therapy (ART).” If this is indeed the case, then what are the ramifications for HBV treatment programs in Africa? In resource-poor settings with overlapping HIV and HBV epidemics one could embed HBV elimination programs in running HIV programs. This should be addressed in brief in the discussion.**

Our comment: We stated in the current discussion that *around 25% of the HBV treatment gap in Zambia has already been addressed by the HIV response. Getting the additional ~120,000 on HBV-active therapy is feasible considering that >800,000 Zambians are currently taking ART for HIV infection.*

We now added the following lines, *Achieving the HIV epidemic control targets (i.e., UNAIDS 95-95-95) will also contribute toward hepatitis B elimination in countries with intersecting* epidemics. HBV activities should ideally be integrated within HIV programs to increase efficiency and reduce costs.

**Reviewer comment: What are the authors’ recommendations in terms of the available guidelines that should be use in Africa based on their analysis/findings? Some comment in this regard would be helpful.**

Our comment: In the revised paper, we added the following comment (page 16, paragraph 3) as to Zambia’s decision around this.

*In its first national hepatitis guidelines, Zambia has adopted WHO recommendations, and where HBV DNA is not available the decision to initiate AVT is based on persistent ALT elevation. Developing low cost, feasible, and accurate criteria on when to treat chronic HBV in Africa is a major priority.*

**Reviewer comment: Figure 1: In the flowchart, the abbreviation “DBS” has been used without the full phrase being used anywhere in the text.**

Our comment: We have removed DBS from the flowchart altogether.

**Reviewer comment: Use of EASL guidelines is reasonable and comparing a variety of guidelines is helpful. However, this highlights the importance of prospective cohort studies to understand treatment and CHB morbidity risks. While EASL guidelines are reasonable they aren’t validated and involve considerable extrapolation in references to Zambia.**

Our comment: We agree with reviewers. There is no gold standard test for ‘who to treat’ for chronic hepatitis B infection and no guideline has been validated, including EASL, AASLD, etc.

**Reviewer comment: I recommend stating what the African validation was for TREAT-B. That is that is was development of a scoring system to approximate EASL criteria. It was not validated to identify individuals at higher risk for CHB complications.**

Our comment: Agree and we have added the following in the revised paper (page 9, paragraph 1), The TREAT-B criterion ia based on categorized ALT level (<20, 20-39, 40-79, and 80+ U/L) and HBeAg status and was validated against EASL.

**Reviewer comment: An important finding of this study is current limitations on CHB research; ie the impact on location and genotype context on scores calculated from biomarkers, age, and fibrosis scores. Whether EASL, WHO, or TREAT-B are the most appropriate for this population is actually unknown. In addition, in all of these settings appropriate treatment duration and criteria for treatment discontinuation remain unclear.**

Our comment: We agree with this point that in the field of CHB there remains fundamental uncertainty over who, when, and how long to treat. We have commented in the discussion that “Developing low cost, feasible, and accurate criteria on when to treat chronic HBV in Africa, as well as how long to treat and when to stop, are major priorities.”

**Reviewer comment: It is unclear which of the authors is affiliated with 8**

Our comment: Thanks for pointing this out. We fixed the affiliations in the revised manuscript.

**Reviewer comment: Incomplete references (eg Shimakawa et al)**

Our comment: We have fixed this in the revised manuscript.
